# The distribution pattern of pelvic lymph nodal metastases in cervical cancer

**DOI:** 10.1007/s00432-023-04810-2

**Published:** 2023-05-26

**Authors:** Shangdan Xie, Jing Zhao, Xintao Wang, Yan Hu, Guannan Feng, Haiyan Zhu, Chao Wang

**Affiliations:** 1 Department of Gynecology, Shanghai First Maternity and Infant Hospital, School of Medicine, Tongji University, 2699 Gaoke West Road, Shanghai, 200092 China; 2 Department of Gynecology, The First Affiliated Hospital of Wenzhou Medical University, Wenzhou, 325035 China; 3 Department of Gynecology, Gusu School, The Affiliated Suzhou Hospital of Nanjing Medical University, Suzhou Municipal Hospital, Nanjing Medical University, Suzhou, 211166 China

**Keywords:** Cervical cancer, Pelvic lymph nodal, Metastases, Distribution

## Abstract

**Purpose:**

Depiction of pelvic lymph node metastasis (LNM) sites among patients with cervical cancer facilitates accurate determination of the extent of dissection and radiotherapy regimens.

**Methods:**

A retrospective study of 1182 cervical cancer patients who underwent radical hysterectomy and pelvic lymph node dissection between 2008 and 2018 was performed. The number of removed pelvic lymph nodes and metastasis status in different anatomical regions was analyzed. The prognostic difference of patients with lymph node involvement stratified by various factors was analyzed by Kaplan–Meier method.

**Results:**

The median number of pelvic lymph nodes detected was 22, mainly from obturator (29.54%) and inguinal (21.14%) sites. Metastatic pelvic lymph nodes were found in 192 patients, with obturator accounting for the highest percentage (42.86%). The patients with lymph node involvement in single site had better prognosis that those in multiple sites. The overall- (*P* = 0.021) (OS) and progression-free (*P* < 0.001) survival (PFS) curves of patients with inguinal lymph node metastases were worse compared to those with obturator site. There was no difference in the OS and PFS among patients with 2 and more than 2 lymph nodes involvement.

**Conclusion:**

An explicit map of LNM in patients with cervical cancer was presented in this study. Obturator lymph nodes tended to be involved. The prognosis of patients with inguinal lymph node involvement was poor in contrast to that with obturator LNM. In patients with inguinal lymph node metastases, clinical staging needs to be reconsidered and extended radiotherapy to the inguinal region needs to be strengthened.

## Introduction

Despite the gradual decline in cervical cancer incidence and mortality with the use of HPV vaccine and cervical screening, mounting studies have shown that patients with lymph node metastasis (LNM) continue to show poor prognosis (Olthof et al. 2022; Sakuragi 2007). The 5-year overall survival (OS) and progression-free survival (PFS) rates of patients with lymph nodes positivity at early stage were significantly lower in contrast to those without lymph node involvement (Saleh et al. 2020). Previously, we reported a significantly shorter PFS in patients with positive lymph nodes than that in negative group (Ji et al. 2022); the metastatic lymph node ratio was independent predictors for PFS (Li et al. 2020). Collectively, lymph node status exerted a pivotal role in the therapy options and prognostic evaluation of cervical cancer. Therefore, accurate evaluation of metastasis status and management these metastatic pelvic lymph nodes is important for the treatment of cervical cancer.

Systematic lymph node dissection allows for a comprehensive evaluation of LNM status, but this approach is accompanied by complications such as lymphedema and lymphocytic cysts. Currently, the technology of sentinel lymph node (SLNs) biopsy is gradually applied to early-stage cervical cancer. According to a large prospective study including 356 Chinese patients with cervical cancer, common sites of SLNs included external iliac (33.38%), obturator (30.08%), common iliac (14.90%), the internal iliac (7.35%), and less frequent sites included presacral (4.33%) and gluteal (4.33%) (Ya et al. 2021). Another study included two prospective database on SLN biopsy and found that the common sites of SLNs showed as following: interiliac/external iliac area (86.3%), common iliac area (8.5%) (Balaya et al. 2020). Clearly, there are significant differences among individuals in the topography of SLNs. Therefore, it is necessary to explore the common metastatic sites of pelvic lymph nodes and to map the metastasis of pelvic lymph nodes in a real-world study. Understanding the topography of LNM in cervical cancer might help in the preoperative evaluation of patients and the development of individualized treatment strategies. In addition, it is an urgent question whether LNM in different regions exhibit a different prognosis.

Hence, the present study aims to describe the mapping of pelvic LNM in cervical cancer and then to analyze the prognostic differences in lymph node involvement in different regions so as to offer a basis for the determination of radiotherapy field or other individual treatment in cervical cancer patients.

## Materials and methods

### Patients

1182 patients were diagnosed with cervical cancer from 2008 to 2018 in the First Affiliated Hospital of Wenzhou Medical University. Radical hysterectomy and pelvic lymph node dissection were performed in all patients. Patients with distant metastasis or uncertain surgical information were excluded. All resected lymph nodes were performed with histopathological examination. All patients signed informed consent and this study was approved by the ethics committee of the First Affiliated Hospital of Wenzhou Medical University.

### Variables

The characteristics utilized in the present study were as follows: age at diagnosis, tumor size, grade, lympho-vascular space invasion (LVSI), anatomical region of removed lymph node, lymph node status (node positive, node negative), FIGO stage (using the FIGO classification of 2009 (Pecorelli 2009)), OS (the time from the beginning of receiving treatment to death), PFS (the time from the beginning of receiving treatment to the onset of progression) and vital status. The cut-off time for follow-up is June 24^th^, 2019.

### Statistical analysis

The data of age at diagnosis, the number of resected and positive lymph nodes (PLNs) were shown as median (P25-P75). The corresponding curves of OS and PFS rates were analyzed by the Kaplan–Meier method, and the comparison among the curves were analyzed through Log-rank test. *P* < 0.05 (two sided) was considered statistically significant.

## Results

### Patient characteristics

Totally, 1182 patients with cervical cancer participated in the present study, and the clinicopathological characteristics of all patients are shown in Table 1. There were 724 (61.25%) patients in FIGO I stage and 458 (38.75%) patients in FIGO II stage. Lymph node metastases were found in 192/1182 (16.24%) patients and 66 patients had ≥ 2 sites of PLNs. LNM was confirmed in 85/724 patients with FIGO I stage and 107/458 patients with FIGO II stage. Lymph nodes tended to be positive in FIGO stage II patients compared with FIGO I patients (*P* < 0.001). All the 1182 patients underwent pelvic lymph node dissection and there was no difference in the number of resected lymph nodes between patients with or without LNM (*P* = 0.530).

**Table 1:** The clinical information of 1182 cervical cancer patients

Variables	Medium	P25-P75
age at diagnosis	52.00	45.00–60.00
The number of removed lymph nodes	22.00	18.00–27.00
The number of positive lymph nodes	2.00	1.00–3.00

Variables	Number of patients	Percent
Tumor size		
< 2 cm	384	32.49%
≥ 2 cm, < 4 cm	441	37.31%
≥ 4 cm	172	14.55%
Unknown	185	15.65%
Grade		
I	505	42.72%
II	424	35.87%
III	74	6.26%
Unknown	179	15.15%
FIGO		
I	724	61.25%
II	458	38.75%
LVSI		
Absent	901	76.23%
Present	268	22.67%
Unknown	13	1.10%
Status of lymph node		
Negative	988	83.59%
Positive	194	16.41%
The number of sites with positive lymph nodes		
1	124	63.92%
≥ 2	70	36.08%

### Anatomical distribution of detected and positive lymph nodes

**Fig. 1:**
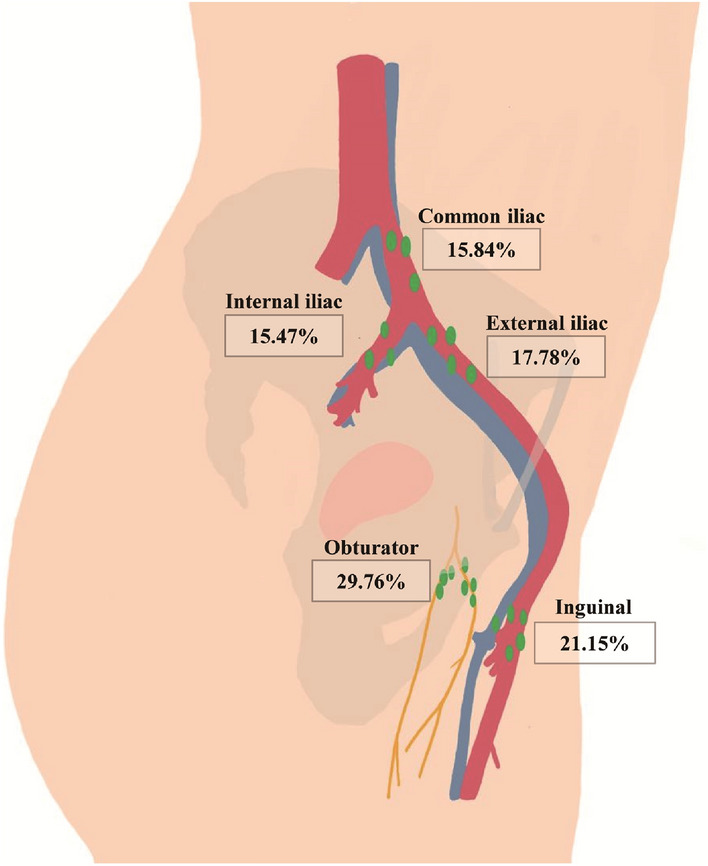
Distribution of resected lymph nodes in 1182 cervical cancer patients

Among 1182 patients with cervical cancer, the median number of lymph nodes detected postoperatively was 22.00 (18.00–27.00). The highest percentage of resected lymph nodes were those within the obturator (29.76%), followed by those from the inguinal site (21.15%), external iliac artery (17.78%), common iliac artery (15.84%), internal iliac artery (15.47%) groups, respectively (Fig. 1).

**Fig. 2:**
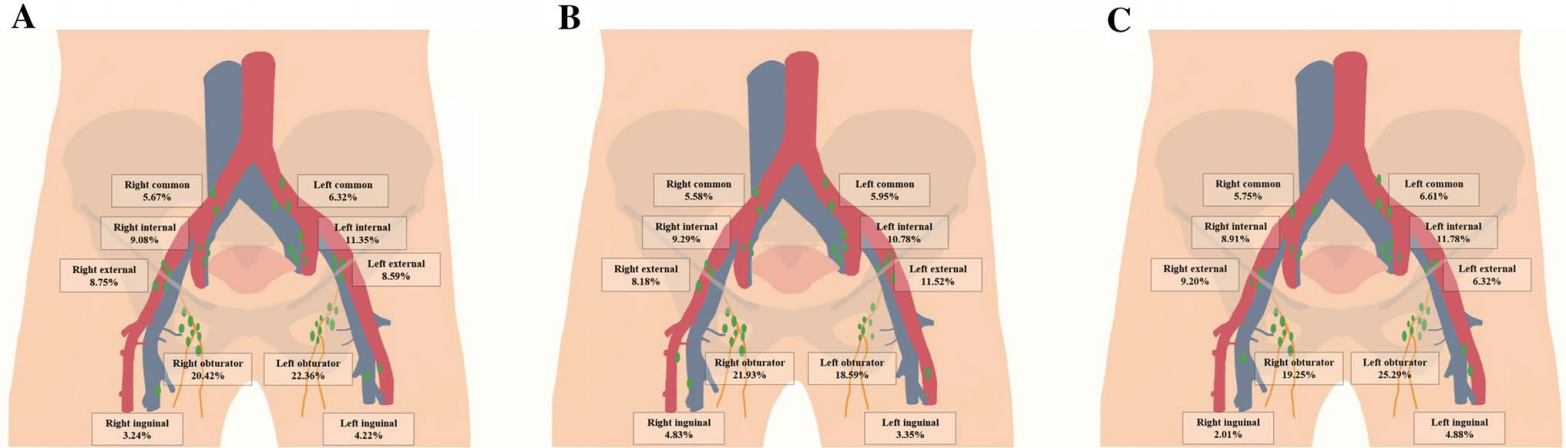
Distributions of positive lymph nodes in patients with different FIGO stage. **A** in 192 patients with FIGO I and II stage; **B** in 85 patients with FIGO I stage; **C** in 107 patients with FIGO II stage

There were 192 cervical cancer patients with PLN, of which 113 (58.85%), 47 (24.48%), 17 (8.85%), 11 (5.73%) and 4 (2.09%) patients had 1, 2, 3, 4 and 5 metastatic sites, respectively. For the number of patients with lymph node involvement in each site, the result showed that obturator group had the largest number, followed by internal iliac group (Table 2). In total, there were 617 PLNs in the 192 patients, and the distribution of LNM is shown in Fig. 2. Overall, the proportion of PLNs in the pelvic cavity were decreased from distant to proximal. The positive ratio of obturator lymph node was the highest and that of inguinal lymph node was the lowest.

**Table 2:** The number of patients with various positive lymph nodes stratified by FIGO stage

Number	FIGO stage		
	I	II	Total
Total patients	724	458	1182
Patients with lymph node metastasis (%)	85 (11.74)	107 (23.36)	192 (16.24)
Left obturator	31	45	76
Right obturator	39	37	76
Left internal iliac	21	32	53
Right internal iliac	18	26	44
Left external iliac	15	17	32
Right external iliac	14	18	33
Left common iliac	10	13	23
Right common iliac	12	7	19
Left inguinal	5	12	17
Right inguinal	10	7	17

### Survival analysis of lymph nodes metastasis stratified by sites

**Fig. 3:**
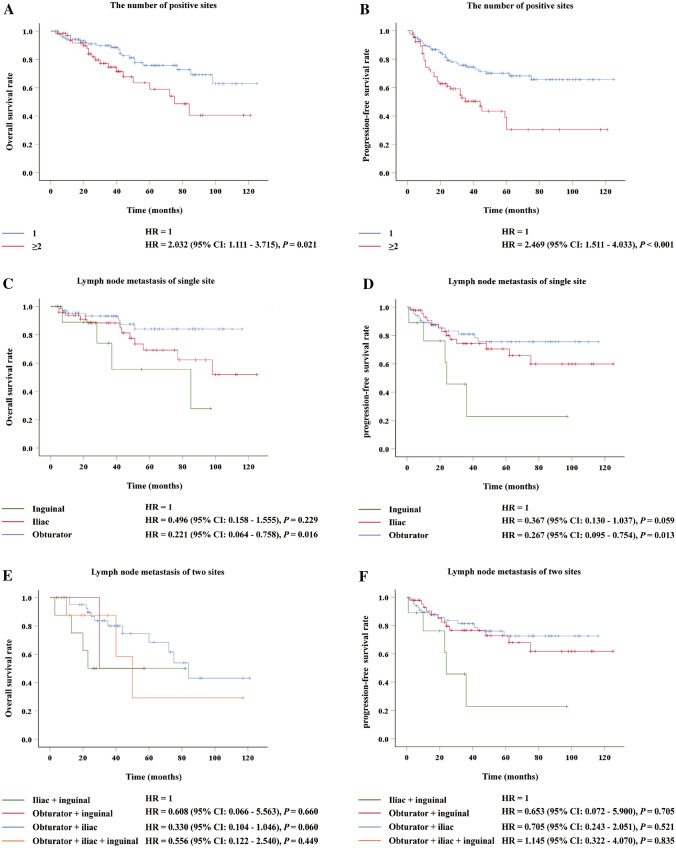
The overall and progression-free survival analysis of cervical patients with lymph node involvement stratified by different factors. (**A**, **B**) by the number of involved sites; (**C**, **D**) by the different single sites of involvement; (**E**, **F**) by the different two sites of involvement

As shown in Fig. 3, the OS and PFS time were higher in patients with single-site lymph node positivity than that in patients with ≥ 2 sites, respectively. Among patients with single-site lymph node involvement, the OS time and PFS time were lower in patients with inguinal involvement than in patients with obturator involvement (*P* < 0.05). There was no difference in prognosis between patients with inguinal metastases and iliac metastases (*P* > 0.05). For patients with multiple sites LNM, there was no difference in the OS or PFS among patients with distinct metastatic sites (*P* > 0.05).

## Discussion

LNM is a common route of cervical cancer metastasis and is an independent element influencing patient survival and recurrence (Feng et al. 2021; Macdonald et al. 2009). The probability of lymph node involvement in patients with FIGO stage I and stage II has been reported to be 0–17% and 12–29%, respectively (Chen et al. 2020; Darai et al. 2008). In our study, 11.88% of patients with FIGO stage I and 23.58% of patients with FIGO stage II showed PLNs, which was consistent with the previous results. The medium dissected number of lymph nodes in the present study was 22 and was similar to previous reports (Olthof et al. 2022; Wang et al. 2020), suggesting that the extent of systematic lymphadenectomy was sufficient in our center, the quality of our procedures was assured, and our results were representative.

The lymph of the uterine cervix drains mainly to the obturator, internal iliac, external iliac, and common iliac lymph nodes, and finally to the presacral and para-aortic lymph nodes (Ercoli et al. 2010). Accordingly, system lymph node dissection mainly includes obturator, internal iliac, external iliac, common iliac, and deep inguinal lymph nodes. Although lymph node involvement in para-aortic site deteriorated the outcomes of cervical cancer patients, the benefit of para-aortic lymph nodes dissection needed further validation (Cho et al. 2020). Owing to the potential complications of lymph node resection, SLN biopsy or selective lymph node dissection was possibly considered as one of the standard therapies for early cervical cancer (Chiyoda et al. 2022; Niu et al. 2022; Poddar and Maheshwari 2021). Consequently, understanding the pattern of LNM contributed to performing lymph node related surgery. In a study of 75 cervical cancer patients, obturator site was the most common area for positive SLNs (Tu et al. 2020). However, another research showed that 44% positive SLNs were detected in external iliac area (Diaz et al. 2011). Due to the inconsistent results, the present study analyzed the pattern of LNM and showed that obturator had the highest proportion of metastasis. In conclusion, obturator was the most common site of LNM. The obturator lymph nodes need more attention during sentinel lymphoscintigraphy and should be removed in lymphadenectomy.

Our previous study showed no difference in the prognosis between patients with unilateral and bilateral lymph node metastases (Li et al. 2020). In the current study, we further analyzed the prognostic differences among cervical cancer patients with different numbers of lymph node metastases. We demonstrated that the patients with metastasized lymph node in single site had better clinical outcomes than those in multiple sites. At present, few studies have focused on the prognostic difference in patients with gynecological tumors with LNM at different sites. Chalif et al*.* demonstrated that PLNs in inguinal region did not deteriorate the outcomes of ovarian patients, while resection to no gross residual disease was related to better prognosis (Chalif et al. 2022). The present study investigated the prognosis of patients with lymph node involvement in distinct sites. Interestingly, we found the patients with lymph nodes positivity in inguinal site had worse outcomes compared with those in obturator site. Due to their specific anatomical position, inguinal lymph nodes did not belong to the pelvic lymph nodes and are less involved in pelvic cancers in contrast to obturator and iliac lymph nodes (Hauspy et al. 2002; Odagiri et al. 2014). Hence, we hypothesize that the prognosis of patients with inguinal LNM may be close to that of patients in the FIGO IV stage, but we need a larger sample size to further confirm our findings. In addition, extended radiation area including inguinal nodes might increase the risk of lower limb edema (Najjari Jamal et al. 2018), the radiotherapy in inguinal site tended not to be performed. Our study suggested that an extended radiation field including inguinal nodes might be helpful for patients with inguinal lymph node metastases. Intensity-modulated radiation therapy (IMRT) is an important adjuvant therapy for cervical cancer in recent years, which could strengthen the treatment of the target field and decrease the damage to the surrounding normal tissues (Lei et al. 2019), and will improve the prognosis of patients with inguinal lymphatic metastases.

Besides, there were some limitations in the present study. Many potential confounding factors might not be excluded in our retrospective study. Next, owing to the deficiency of prospective design, SLN biopsy was not analyzed in the present study. Finally, the patients were recruited from only one center and the number of participants was limited, so the results in our study needed to be validated by more subjects from multiple centers.

## Conclusion

 Our study mapped pelvic LNM in patients with cervical cancer and showed that obturator was the most common site of metastatic. Therefore, more attention should be paid to obturator when performing a systematic lymphadenectomy and SLN. Patients with inguinal metastases have a worse prognosis than those with obturator metastases. In patients with inguinal LNM, clinical staging needs to be reconsidered and extended radiotherapy to the inguinal region needs to be strengthened.

## Data Availability

The data that support the findings of this study are available from the corresponding author on reasonable request.
